# Oral Administration of Sitagliptin Activates CREB and Is Neuroprotective in Murine Model of Brain Trauma

**DOI:** 10.3389/fphar.2016.00450

**Published:** 2016-12-01

**Authors:** Brian DellaValle, Gitte S. Brix, Birgitte Brock, Michael Gejl, Jørgen Rungby, Agnete Larsen

**Affiliations:** ^1^Department of Biomedicine/Pharmacology, Aarhus UniversityAarhus, Denmark; ^2^Centre of Medical Parasitology, Department of Clinical Microbiology, Copenhagen University HospitalCopenhagen, Denmark; ^3^Department of Clinical Biochemistry, Aarhus University HospitalAarhus, Denmark; ^4^Department of Endocrinology, Bispebjerg University HospitalCopenhagen, Denmark

**Keywords:** GLP-1, traumatic brain injury, TBI, sitagliptin, liraglutide, CREB, GIP, DPP-IV

## Abstract

**Introduction:** Traumatic brain injury is a major cause of mortality and morbidity. We have previously shown that the injectable glucagon-like peptide-1 (GLP-1) analog, liraglutide, significantly improved the outcome in mice after severe traumatic brain injury (TBI). In this study we are interested in the effects of oral treatment of a different class of GLP-1 based therapy, dipeptidyl peptidase IV (DPP-IV) inhibition on mice after TBI. DPP-IV inhibitors reduce the degradation of endogenous GLP-1 and extend circulation of this protective peptide in the bloodstream. This class has yet to be investigated as a potential therapy for TBI.

**Methods:** Mice were administrated once-daily 50 mg/kg of sitagliptin in a Nutella® ball or Nutella® alone throughout the study, beginning 2 days before severe trauma was induced with a stereotactic cryo-lesion. At 2 days post trauma, lesion size was determined. Brains were isolated for immunoblotting for assessment of selected biomarkers for pathology and protection.

**Results:** Sitagliptin treatment reduced lesion size at day 2 post-injury by ~28% (*p* < 0.05). Calpain-driven necrotic tone was reduced ~2-fold in sitagliptin-treated brains (*p* < 0.001) and activation of the protective cAMP-response element binding protein (CREB) system was significantly more pronounced (~1.5-fold, *p* < 0.05). The CREB-regulated, mitochondrial antioxidant protein manganese superoxide dismutase (MnSOD) was increased in sitagliptin-treated mice (*p* < 0.05). Conversely, apoptotic tone (alpha-spectrin fragmentation, Bcl-2 levels) and the neuroinflammatory markers IL-6, and Iba-1 were not affected by treatment.

**Conclusions:** This study shows, for the first time, that DPP-IV inhibition ameliorates both anatomical and biochemical consequences of TBI and activates CREB in the brain. Moreover, this work supports previous studies suggesting that the effect of GLP-1 analogs in models of brain damage relates to GLP-1 receptor stimulation in a dose-dependent manner.

## Introduction

Traumatic brain injury (TBI) is a major cause of mortality and morbidity across the globe. Severe TBI cases are fatal for over one third of patients and an estimated 60% are burdened with unfavorable outcomes (Rosenfeld et al., 2012; DeKosky et al., 2013). Moreover, secondary inflammation, oxidative stress and cerebral edema after injury can contribute to an increased lesion size and potential for neurodegenerative changes in the brain (Alahmadi et al., 2010; Rosenfeld et al., 2012; DeKosky et al., 2013). We have previously shown that the injectable glucagon-like peptide-1 (GLP-1) analog, liraglutide, significantly improved the outcome in mice after severe brain trauma (DellaValle et al., 2014). In this study we assess the effect of oral treatment with a different class of GLP-1-based therapy, sitagliptin- a dipeptyl peptidase IV (DPP-IV) inhibitor- on mice after brain injury. This class of compounds has not yet, to our knowledge, been investigated in a model of TBI.

DPP-IV inhibitors reduce the degradation of endogenous GLP-1 and extend circulation of this cytoprotective peptide in the bloodstream (Drucker and Nauck, 2006). In this regard we investigated prolonged exposure to endogenous GLP-1 as a therapeutic against severe TBI. In our previous work, GLP-1 agonism with a DPP-IV-resistant analog, liraglutide, reduced lesion size and broad-spectrum cell death (DellaValle et al., 2014), and liraglutide reduced edema in a cortical impact model of TBI in rats (Hakon et al., 2015). In addition, the GLP-1 receptor agonist exendin-4 has been shown to improve behavior (Rachmany et al., 2013) after mild TBI in mice. Furthermore, DPP-IV inhibition prolongs activity of other neuroactive peptides such as glucose-dependent insulinotrophic polypeptide (GIP)- a peptide recently shown to improve cognitive deficits after mild traumatic brain injury in rats (Yu et al., 2016).

The neuroprotective potential of GLP-1 agonism is well-described in animal models of neurodegeneration, ischemia, neuroinflammation, and cognitive impairment (Candeias et al., 2015). Moreover, DPP-IV inhibition has been shown to have similar neuroprotective effects as GLP-1 agonism in rodent models of cognitive decline (Pintana et al., 2013; Pipatpiboon et al., 2013; Gault et al., 2015; Tsai et al., 2015) and cerebral ischemia (Yang et al., 2013; Ma et al., 2015) possibly, in part due to suppression of oxidative stress (Pintana et al., 2013; El-Sahar et al., 2015; Gault et al., 2015; Tsai et al., 2015). However, the mechanism of this protective effect is still poorly understood.

GLP-1 activates the protective cAMP response element binding protein (CREB) system (Jhala et al., 2003; Drucker, 2006). In our previous work, we established that the GLP-1 analog liraglutide activates CREB in the brain *in vivo* and this activation results in increased production of neuroprotective proteins regulated by CREB (DellaValle et al., 2014)- many of which are related to suppression of oxidative stress. This response in the brain was driven through the GLP-1 receptor (DellaValle et al., 2014). Interestingly, this activation is nevertheless absent in healthy mice treated with liraglutide despite an increase in cerebral cAMP levels (DellaValle et al., 2016). These data suggest a complex and dynamic interaction between GLP-1 signaling and the activation of CREB in the brain. Indeed, DPP-IV inhibition has been shown to increase CREB activation in pancreatic islets (Samikannu et al., 2013) and myocardial tissue (Ye et al., 2010; Ihara et al., 2015) though it is unclear whether this effect is present in the brain. We were therefore interested in the potential for DPP-IV inhibitors to activate this protective pathway.

In this investigation we utilize a severe brain trauma model to test the hypothesis that oral inhibition of DPP-IV would provide a protective effect after TBI and activate the CREB system in the brain.

## Materials and methods

Female C57Bl6/j mice (Taconic, Lille Skensved, Denmark) aged 6–8 weeks were kept under standard conditions with food/water access *ad libitum*. Studies were conducted to minimize suffering and, in accordance with predefined humane endpoints, were approved by the Danish Animal Inspectorate according to the license 2012-15-2934-00448 and are in accord with the National Institutes of Health guidelines.

Mice were weighed and randomly separated into vehicle and sitagliptin groups. Mice were administrated once-daily 50 mg/kg of sitagliptin in a 0.1 mL Nutella® ball or Nutella® alone throughout the study, beginning 2 days before TBI. This dose in mice on a once-daily, oral regime has been shown to increase concentrations of GLP-1 in the brain, have no adverse events and to have no effect on weight or energy intake (Gault et al., 2015). TBI was induced blinded to the treatment groups with a stereotactic cryogenic lesion as described and characterized in detail in (DellaValle et al., 2014). Two-percent lidocaine was applied to the scalp 20 min before the procedure. Under isoflurane anesthesia, a contra-lateral skin incision was made and a stereotactic lesion was induced with a liquid nitrogen-acclimatized, flat cryoprobe (thermal conductivity ~ 120 W/mK, 3.0 mm diameter, CryoPro; Cortex, Hadsund, Denmark) applied to the skull 1.5 mm lateral and 1.5 mm posterior to the bregma for 90 sec under force of gravity (0.39 N). The incision was stapled and lidocaine was applied at the incision site. Animals were under anesthesia for ~7 min. Treatment continued once-daily for 2 days post-TBI and signs of distress and complications were monitored.

At day 2 post-lesion, animals were anesthetized with Hypnorm/Dormicum, weighed, and transcardially perfused with heparinized saline (0.9%). Brains for immunoblotting (Veh, *n* = 9, sitagliptin, *n* = 10) were isolated, the brainstem, cerebellum, and olfactory bulbs removed and the remaining cerebrum split into ipsilateral and contralateral hemispheres and the ipsilateral hemisphere was snap frozen in liquid nitrogen and stored at −80°C for immunoblotting.

### Lesion size determination

Brains for lesion size determination (veh = 15, sitagliptin = 11) were sectioned (1 mm) in a coronal matrix (BSMAS001-1; Zivic Instruments, Pittsburgh, PA, USA), and incubated in 1% 2,3,5-triphenyltetrazolium (TTC, in saline; Sigma-Aldrich, Brøndby, Denmark) for 30 min at 37°C. TTC is a functional mitochondrial stain. Planimetry was performed with ImageJ software (National Institutes of Health, Bethesda, MD, USA) blinded to treatment groups comparing ipsilateral and contralateral hemispheres. Sections where lesions extended into adjacent sections but did not pass entirely through the 1 mm were excluded due to imprecision of the depth dimension.

### Immunoblotting

The ipsilateral hemisphere of the cerebrum was homogenized with protease+phosphatase inhibitors (Roche, complete mini, DK Phosphosafe; Millipore), protein content quantified, aliquoted, and stored at −22°C. Immunoblotting was optimized and performed with standard Western blot principles on the ipsilateral hemisphere encompassing the lesion site. Homogenates were reduced, heated, and loaded at 25–50 μg into precast polyacrylamide gels [12% or 4–12% (α-spectrin) (NuPAGE; Life Technologies, Naerum, Denmark)] and gels run in MES buffer and transferred to polyvinylidene difluoride membrane. Membranes were washed in tris-buffered saline (TBS), and blocked in 5% skim milk powder or bovine serum albumin + TBS-(0.01% Tween) for 1 h at room temperature. Primary antibodies were applied in appropriate blocking solution overnight at 4°C and are listed in detail in Table 1. Secondary antibodies were applied in appropriate blocking solution: Horseradish peroxidase-conjugated-conjugated anti-rabbit/anti-mouse (Dako, Glostrup, Denmark) at 1:2000 and 1:3000, respectively, for 1 h at room temperature. Membranes were incubated in SuperSignal Femto substrate (34095; Thermo Scientific) and exposed with a CCD camera (Bio-Rad Chemidoc XRS imager, Copenhagen, Denmark). Images were quantified with ImageJ and reported relative to housekeeping protein, glyceraldehyde 3-phosphate dehydrogenase (GAPDH).

**Table 1:** **Antibody origin and protocol**.

**Antigen**	**Company, product #**	**Molecular mass (kDa)**	**Blocking solution**	**Dilution**
α-Spectrin	Millipore, MAB 1622	150/145/120	5% SMP TBS-T	1 to 1000
CREB	Cell signaling, 9197	43	5% SMP TBS-T	1 to 1000
pCREB	Millipore, 06-519	43	5% BSA TBS-T	1 to 1000
MnSOD	Millipore, 06-984	24	5% SMP TBS-T	1 to 1000
Bcl-2	Cell signaling, 2876S	28	5% BSA TBS-T	1 to 1000
IL-6	Abcam, ab6672	22	5% BSA TBS-T	1 to 500
Iba-1	WAKO, 016-20001	17	5% BSA TBS-T	1 to 1000
GAPDH	Millipore, MAB 374	39	5% BSA or SMP TBS-T	1 to 10000

*Detailed description of antibodies, origin of purchase, molecular weight quantified, blocking solution, and dilutions used for immunoblotting*.

### Immunoblotting of homogenates from previous study

Ipsilateral brain homogenates from a previous study (DellaValle et al., 2014) were analyzed with an identical protocol. Brains were prepared from the same time point post-lesion (day 2), from the same TBI model, and the same immunoblotting protocol for determining manganese superoxide dismutase (MnSOD) levels. Mice in the previous study were treated with GLP-1 analog, liraglutide, or vehicle (PBS) twice-daily beginning directly after TBI induction.

### Data analysis

Data sets were tested for normality (Shapiro–Wilk) and equal variance before statistical analyses were performed. Non-normal data was log transformed. If data remained non-normal and/or variances were unequal after transformation, non-parametric rank statistics were applied. Student's *t*-test: Lesion size, weight change, and immunoblotting (Mann-Whitney: α-spectrin fragment data). A *P*-value of <0.05 was reported as statistically significantly different. Data are presented as mean ± S.E.M for normal data and median ± interquartile range for non-normal data.

## Results

The weight of mice before and after the investigation did not change with sitagliptin treatment: Mean weight difference±S.E.M: Vehicle: −0.51±0.19g vs. −0.71±0.17g; *p* = 0.4).

**Figure 1:**
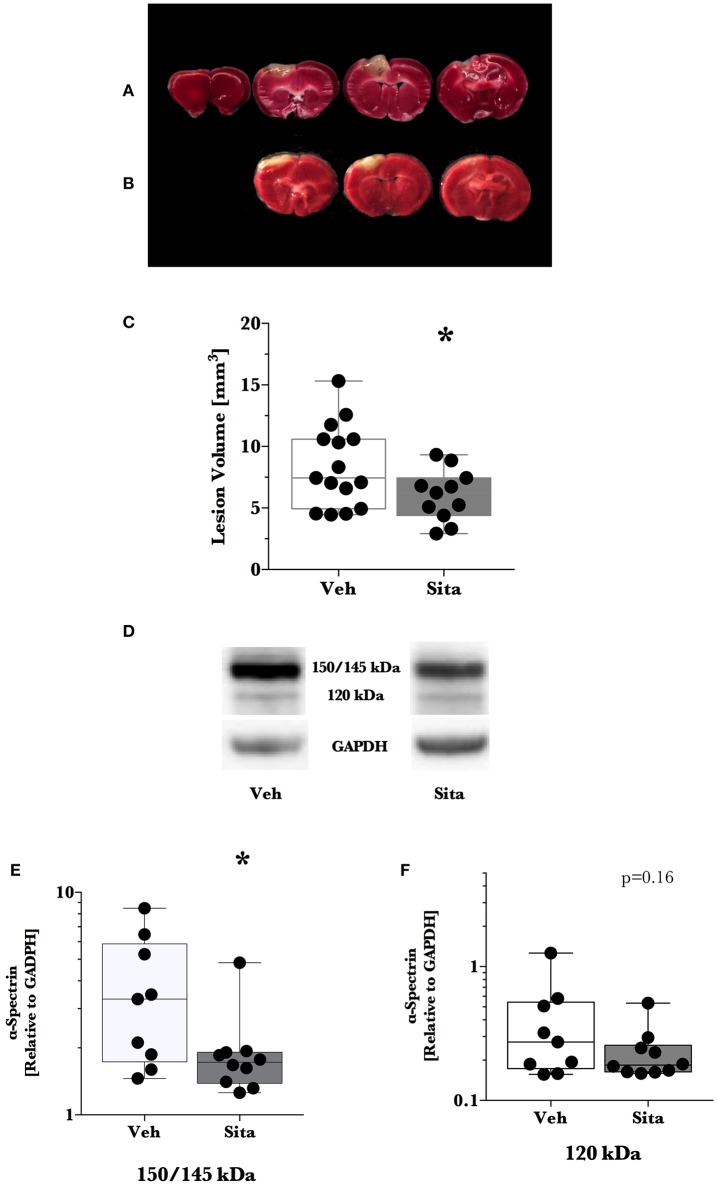
**Lesion size determination and cell death signaling**. Mice were treated once-daily with vehicle (Veh) or sitagliptin (Sita) for 2 days. Traumatic brain injury (TBI) was induced blinded to the treatment arms and thereafter treatment continued once-daily for a subsequent 2 days post-TBI. Brains were sliced (1 mm), stained with 1% 2,3,5-triphenyltetrazolium, and quantified with planimetry **(A–C)** or the cerebrum of ipsilateral hemisphere was isolated for immunoblotting **(D–F)**. Representative **(A)** Veh- and **(B)** Sita-treated injury volume after staining. **(C)** Box-whisker plot presenting lesion volume (mm^3^) at day 2 for Veh- (white) and Sita- (black) treatment arms. Adjacent sections with lesioned tissue that did not pass entirely through the 1 mm section were not quantified. Sita treatment significantly reduced lesion size volume. All data points are presented with box (25 to 75th percentile; line = median) and whiskers: min and max; Veh, *n* = 15, Sita, *n* = 11. Alpha-spectrin fragmentation was quantified with immunoblotting of the ipsilateral hemisphere (representative blotting lane: **D**) where bands at 150+145 kDa **(E)** and 120 kDa **(F)** were analyzed separately representing necrotic and apoptotic signaling, respectively. Signals are reported relative to the housekeeping signal of GAPDH. All data points are reported with box (25 to 75th percentile; line = median) and whiskers (min and max). Data in **(E,F)** is non-parametric and is presented with a log10 y-axis. Necrotic signaling was reduced by Sita treatment whereas apoptotic signaling was not significantly reduced. Veh, *n* = 9, Sita, *n* = 10. ^*^ *p* < 0.05.

We predetermined that lesion volume 2 days after TBI would be the primary outcome. Sitagliptin treatment resulted in a ~28% decrease in lesion size volume (6.04±0.63 mm^3^ vs. 8.41±0.86 mm^3^; *p* < 0.05; *n* = 11, 15; Figures 1A–C).

**Figure 2:**
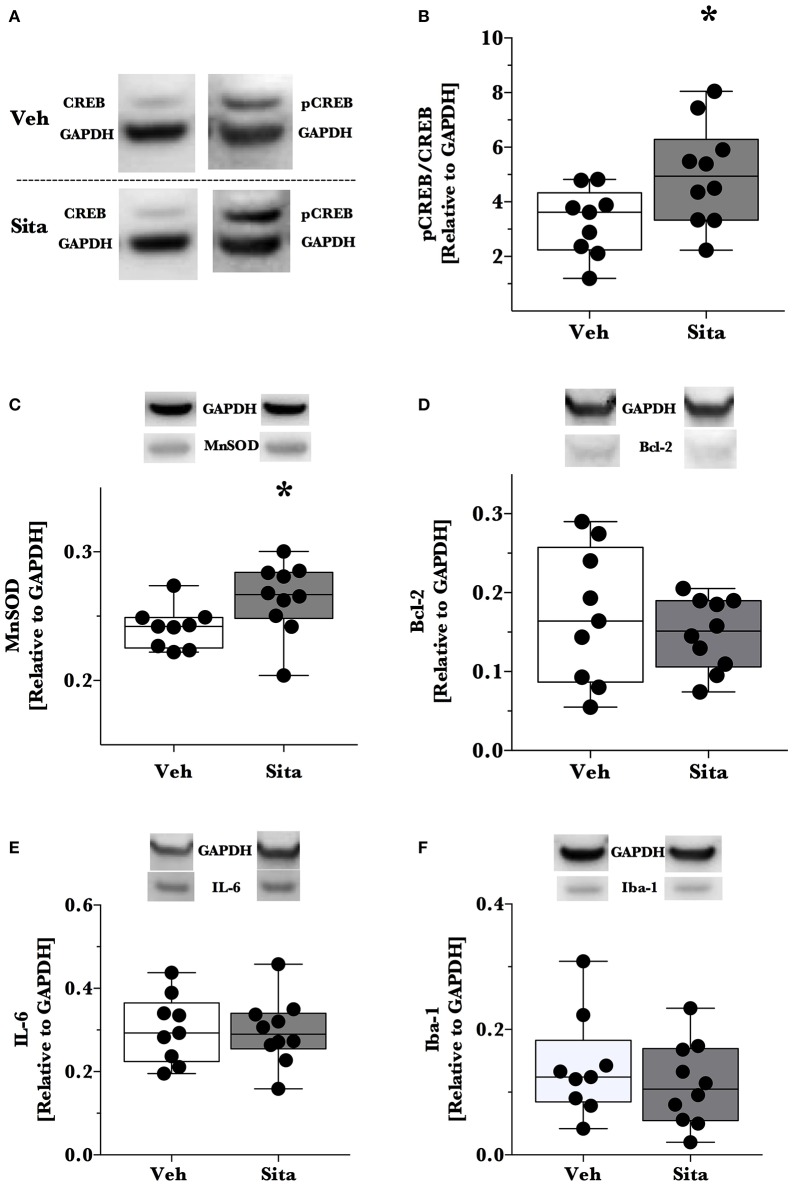
**Activation of CREB, downstream effector proteins and marker of microglial activation**. Traumatic brain injury (TBI) animal brains were excised at day 2 post-lesion, cerebrum isolated, and the ipsilateral hemisphere processed for immunoblotting. Brains were thereafter probed for CREB and phosphorylated CREB_Ser133_ **(A,B)**, **(C)** MnSOD, **(D)** Bcl-2, **(E)** IL-6 and Iba-1 **(F)** and are reported relative to the housekeeping signal of GAPDH. Representative blotting lanes are provided in **(A–F)** for respective targets. Molecular weights of quantified bands are provided in Table 1. All data points are reported with box (25 to 75th percentile; line = median) and whiskers (min and max). Sita treatment significantly activates the CREB system and slightly increases the levels of CREB-regulated MnSOD. Bcl-2 and IL-6 levels were not affected by Sita treatment and activated microglial marker Iba-1 did not change with treatment. Veh, *n* = 9, Sita *n* = 10. ^*^ *p* < 0.05.

Thereafter, we assessed cell death signaling in the brain via α-spectrin fragmentation (Figures 1D–F). α-Spectrin, a 260 kDa protein, is cleaved into different fragments by activation of necrotic (primarily calpain-driven) and apoptotic (primarily caspase-driven) pathways at the cellular level. As seen in Figure 1, necrotic signaling in the ipsilateral hemisphere of sitagliptin-treated mice was significantly reduced ~2-fold relative to vehicle-treated mice after TBI (*p* = 0.043, Mann-Whitney). Conversely, apoptotic signaling was not significantly reduced (*p* = 0.16). With this observed reduction in lesion size and broad reduction in necrotic signaling, we were interested if the neuroprotective CREB system was significantly activated in animals receiving sitagliptin treatment. We predefined “activation” as the ratio of phosphorylation of CREB to unphosphorylated CREB (pCREB/CREB). Indeed, the CREB system in the ipsilateral hemisphere of sitagliptin-treated mice were ~1.5-fold higher than vehicle–treated mice (*p* = 0.029; Figures 2A,B). Our previous work suggested GLP-1 receptor agonism provides antioxidant support after TBI. In this regard we assessed the protein levels of the CREB-regulated mitochondrial antioxidant MnSOD. We detected a small but significant (*p* = 0.042) increase in MnSOD in sitagliptin-treated animals (Figure 2C). From this finding, we endeavored to assess MnSOD levels in ipsilateral brain homogenates from mice at day 2 post-lesion from our previous study treated with liraglutide (DellaValle et al., 2014). Indeed, similar to our findings in lesion size and CREB, we found a more pronounced effect with liraglutide: A ~1.6–fold increase in MnSOD at day 2 post lesion (*p* < 0.001; Supplemental Figure 1).

Although the CREB system is significantly activated in sitagliptin-treated animals, we did not detect a significant difference the apoptotic tone. We therefore expanded on this finding by assessing the levels of the CREB-regulated anti-apoptotic protein, Bcl-2 (Figure 2D). In line with this- and our previous work with TBI and liraglutide (DellaValle et al., 2014), there was no difference in Bcl-2 levels between treatment groups (*p* = 0.48).

In this previous work, IL-6 was significantly reduced by liraglutide treatment. Moreover, DPP-IV-inhibitors may have effect on the peripheral and endothelial inflammatory response. On this basis, we probed for IL-6 content in the brain and the activated microglial marker Iba-1 (Glushakov et al., 2016). Sitagliptin did not have an effect on the protein levels of IL-6 or Iba-1 (Figures 2E,F).

## Discussion

In this study, we exploit the therapeutic potential of modulating endogenous GLP-1 responses using DPP-IV inhibition, a GLP-1 degradation inhibitor with the additional benefits of neuroprotective activity of other neuroactive peptides such as GIP (Drucker and Nauck, 2006). We found that oral administration of sitagliptin resulted in similar effects as the GLP-1 analog, liraglutide, albeit to a lesser extent. This is to be expected as the concentration of GLP-1 in the brain is likely to be considerably lower than with liraglutide treatment (Holst et al., 2008) and moreover, the production of GLP-1 itself is regulated by the endogenous production cycle of each individual animal. However, the oral administration of DPP-IV inhibitors is an attractive feature in comparison to other GLP-1 agonists (often delivered subcutaneously); a feature that is presently being explored by developers of oral GLP-1 receptor agonists.

This study further confirms the ability of GLP-1-based therapies to reduce lesion size after TBI and activate the neuroprotective CREB system in the brain. Necrotic tone in the damaged hemisphere was reduced by sitagliptin treatment and apoptotic tone was not- although it tended to be reduced. This reduction in tissue damage may be associated with CREB-regulated protective proteins that we previously described to be involved in GLP-1 analog treatment (DellaValle et al., 2014): Brain-derived neurotrophic factor, peroxisome proliferator-activated receptor alpha, and neuroglobin- however, other CREB-regulated proteins are also protective. MnSOD is a mitochondrial antioxidant (Galeotti et al., 2005) involved in mitigating secondary tissue damage. This protein was slightly increased by sitagliptin treatment, and upon further analysis of tissue from our previous study, was increased to a larger extent with liraglutide treatment. This antioxidant is a major detoxifying protein against reactive oxygen species in the mitochondria, suggesting that mitochondrial health is improved with GLP-1 receptor agonism. This is further supported by the increased TTC staining in the lesion size determination- effectively a mitochondrial vitality stain. This supports previous work where reactive oxygen species were reduced after TBI and two previously mentioned proteins involved in mitochondrial health, peroxisome proliferator-activated receptor alpha, and neuroglobin, were increased by GLP-1 analog treatment (DellaValle et al., 2014). Moreover, in addition to the CREB system, GLP-1 receptor activation is also thought to be cytoprotective in the brain through the Akt pathway (Kimura et al., 2009; Pipatpiboon et al., 2013; Sharma et al., 2014).

Recently, we investigated GLP-1 analog treatment in a cerebral complication of malaria, where cell death and inflammation is present but frank oxidative stress is not directly implicated (DellaValle et al., 2016). In this model, GLP-1 agonism with liraglutide did not activate the CREB system and was not protective. Moreover, despite increasing intracellular cAMP in healthy animals, liraglutide did not activate CREB. These data suggest that CREB activation is an important mechanism for GLP-1 protection in the brain, especially in diseases where oxidative stress is a clear component of the pathogenesis.

Recent work by Knudsen et al. (2016) with a fluorescent-linked liraglutide compound showed the distribution of internalized liraglutide in the healthy mouse brain after drug delivery and thus, represents a surrogate for the distribution of GLP-1 receptor activation from the periphery. Although there is little accumulation of liraglutide in the cortical tissue associated with this work in that study in the healthy brain, it should be stressed that access of GLP-1 into the brain parenchyma is likely improved after trauma due to a disrupted blood-brain barrier and, more importantly, receptor density, and cell expression profiles may be altered in response to pathology. We suggest that a possible target of GLP-1 agonism after TBI may be, in part, through astrocytic up-regulation of GLP-1 receptor at the lesion border. This has been suggested in 1999 by work from Chowen et al. after a penetrating cortical lesion (Chowen et al., 1999). We have also previously reported that neuroglobin- a GLP-1-promoting (DellaValle et al., 2014), CREB-regulated (De Marinis et al., 2013) protein- is up-regulated in a similar population of astrocytes at the border region of a cortical lesion (DellaValle et al., 2010). Indeed, astrocytes have an important role in protection after injury in the brain (Sofroniew, 2009) and liraglutide activates CREB in cultured astrocytes and protects them from oxidative damage (Bao et al., 2015). Other possible mechanisms for the effects of GLP-1 in the brain remain to be explored. Finally, the activation of antioxidants may suggest an increased fuel combustion and we have recently demonstrated in human studies that a perturbed cerebral glucose metabolism can be corrected by GLP-1 in both health and neurodegenerative disease (Lerche et al., 2008; Gejl et al., 2012, 2014, 2016).

A growing body of evidence suggests that GLP-1-based therapies may have potential as therapeutics after TBI and in other neurodegenerative diseases. In this study we demonstrate, for the first time, that DPP-IV inhibitors can be considered- in addition to direct GLP-1 receptor agonists- in this emerging treatment strategy for TBI.

## Ethics statement

Danish Animal Inspectorate according to the license 2012-15-2934-00448.

## Author contributions

BD: Concept, design, conducted experiments, analyzed and interpreted data, drafted work, revised, final approval. GB: Design, conducted experiments, revised work, Final approval. MG: Concept, revised work, final approval. BB: Concept, revised work, final approval. JR: Concept, design, revised work, final approval. AL: Concept, design, revised work, final approval.

## Funding

The authors thank A. P. Møller foundation for the financial support. The supplemental material is new, original work on tissue from a study conducted when BD was partly funded by Novo Nordisk Foundation, and Novo Nordisk.

### Conflict of interest statement

BD: No conflict of interest regarding DPP-IV inhibition and TBI. Supplemental material is new, original work on tissue from a study conducted when BD was partly funded by Novo Nordisk. Independence of the researchers was secured through an intellectual property rights agreement approved by the legal department of University of Copenhagen. In accordance with the IPR, the company had no influence on the experiments, content of the manuscript or the decision to publish. This does not alter the authors' adherence to all policies on sharing data and materials. GB, BB, MG, JR, and AL declare that the research was conducted in the absence of any commercial or financial relationships that could be construed as a potential conflict of interest.

## Supplementary material

Supplementary Figure 1 **Immunoblotting for MnSOD levels from an independent study involving treatment with GLP-1 analog, liraglutide**. Upon detecting a similar pattern in traumatic brain injury (TBI) mice treated with sitagliptin, we endeavored to analyze brain homogenates prepared in an identical manner from a previous study in the same TBI model for MnSOD levels. The treatment groups were: vehicle (PBS) and the long-lasting glucagon-like peptide-1 (GLP-1) analog, liraglutide. TBI was induced in the same manner. Directly after TBI induction, mice were randomly selected for twice-daily subcutaneous injections of either PBS or liraglutide. At day 2 post-lesion, the cerebrum was isolated and ipsilateral hemisphere processed for immunoblotting in the same manner. Brain homogenates were then probed for MnSOD levels and are reported relative to the housekeeping signal of GAPDH. Representative blotting lanes are provided and all data points are reported with box (25 to 75th percentile; line = median) and whiskers (min and max). Similar to mice treated with sitagliptin, MnSOD levels were increased in mice treated with liraglutide. ^***^*p* < 0.001.

## Abbreviations

Bcl-2: B-cell lymphoma protein-2
CREB: cAMP response element binding protein
DK: Denmark
DPP-IV: Dipeptyl peptidase IV
GAPDH: Glyceraldehyde 3-phosphate dehydrogenase
GIP: Glucose-dependent insulinotrophic polypeptide
GLP-1: Glucagon-like peptide-1
MnSOD: Manganese superoxide dismutase
TBI: Traumatic brain injury
TTC: 2,3,5-triphenyltetrazolium.
