# Supplementary figures and images for: Oral Administration of Sitagliptin Activates CREB and Is Neuroprotective in Murine Model of Brain Trauma
Source: Front Pharmacol. 2016 Dec 1;7:450. doi: 10.3389/fphar.2016.00450 (PMC5130988; doi:10.3389/fphar.2016.00450)

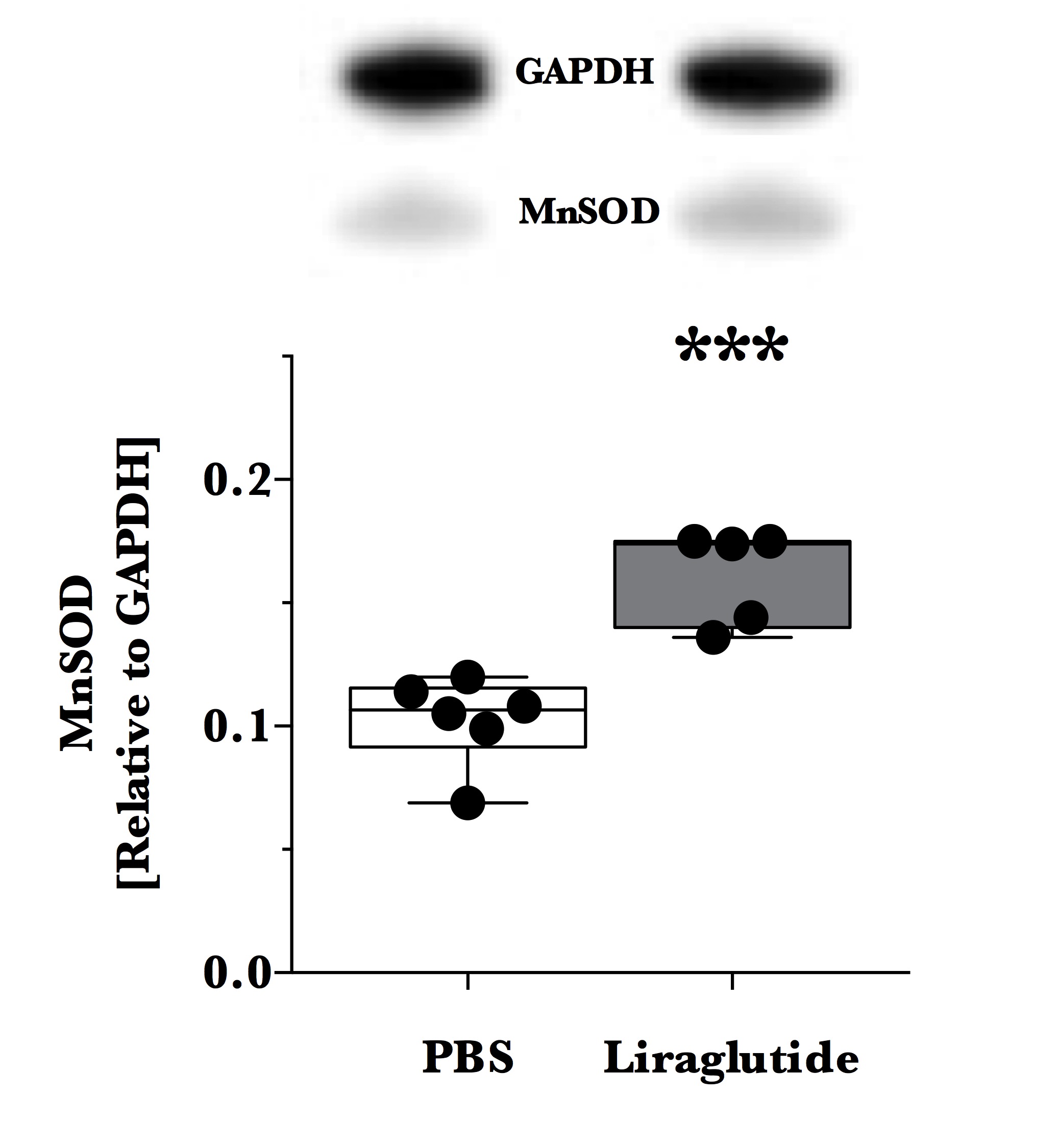

Supplement: Supplementary Figure 1 — Immunoblotting for MnSOD levels from an independent study involving treatment with GLP-1 analog, liraglutide. Upon detecting a similar pattern in traumatic brain injury (TBI) mice treated with sitagliptin, we endeavored to analyze brain homogenates prepared in an identical manner from a previous study in the same TBI model for MnSOD levels. The treatment groups were: vehicle (PBS) and the long-lasting glucagon-like peptide-1 (GLP-1) analog, liraglutide. TBI was induced in the same manner. Directly after TBI induction, mice were randomly selected for twice-daily subcutaneous injections of either PBS or liraglutide. At day 2 post-lesion, the cerebrum was isolated and ipsilateral hemisphere processed for immunoblotting in the same manner. Brain homogenates were then probed for MnSOD levels and are reported relative to the housekeeping signal of GAPDH. Representative blotting lanes are provided and all data points are reported with box (25 to 75th percentile; line = median) and whiskers (min and max). Similar to mice treated with sitagliptin, MnSOD levels were increased in mice treated with liraglutide. ***p < 0.001. [file Image1.JPEG]
